# *Panax notoginseng* saponins alleviates metabolic dysfunction-associated steatotic liver disease in mice by influencing Nrf2/MT2

**DOI:** 10.3389/fphar.2026.1874424

**Published:** 2026-06-29

**Authors:** Lu Liu, Yifan Bai, Ximeng Liu, Yiwen Zhang, Jia Li, Yilin Wang, Xinying Cao, Hanwei Li, Jun Wang, Shushu Tan, Shuihua Wu, Yige Wang, Yilin Li, Jinxin Miao

**Affiliations:** 1 Academy of Chinese Medical Sciences, Henan University of Chinese Medicine, Zhengzhou, China; 2 Institute of Chinese Materia Medica, China Academy of Chinese Medical Sciences, Beijing, China; 3 Guangxi Wuzhou Pharmaceutical (Group) Co. Ltd., Wuzhou, China; 4 Acupuncture and Massage College, Henan University of Chinese Medicine, Zhengzhou, China

**Keywords:** metabolic dysfunction-associated steatotic liver disease, metallothionein-2, nuclear factor erythroid 2-related factor 2, oxidative stress, *Panax notoginseng* saponins

## Abstract

**Background:**

Metabolic dysfunction-associated steatotic liver disease (MASLD) is a metabolic liver injury driven largely by oxidative stress. The nuclear factor erythroid 2-related factor 2 (Nrf2) pathway plays a central role in antioxidant defense, and metallothionein-2 (MT2) is a potential downstream target. *Panax notoginseng* saponins (PNS) exhibit anti-inflammatory and antioxidant properties, but their effects on MASLD *via* Nrf2/MT2 signaling remain unclear. This study aimed to investigate whether PNS exerts protective effects against MASLD through Nrf2/MT2 signaling pathway.

**Methods:**

Apolipoprotein E knockout mice were fed a high-fat diet (HFD) to establish MASLD model with PNS treatment. Hematoxylin and Eosin Staining (HE) and Oil Red O staining were conducted. Levels of triglyceride (TG), total cholesterol (TC), aspartate aminotransferase (AST), alanine aminotransferase (ALT), interleukin-6 (IL-6) and tumor necrosis factor-α (TNF-α) were measured. RNA sequencing was conducted, and expression of Nrf2, MT2 was measured. Molecular docking and surface plasmon resonance (SPR) assays were conducted to evaluate interactions between major saponins of PNS and MT2 protein.

**Results:**

PNS alleviated lipid accumulation and liver injury in the MASLD model. PNS significantly alleviated oxidative stress and inflammation potentially through Nrf2/MT2 in MASLD mice. Molecular docking revealed that the five major saponins of PNS exhibited favorable binding affinities to MT2. SPR results further demonstrated that ginsenoside Rd may have the most stable binding conformation with MT2.

**Conclusion:**

Our study demonstrates that PNS effectively reduces oxidative stress and inflammation in MASLD, potentially through Nrf2/MT2 pathway, and ginsenoside Rd exhibits favorable binding to MT2. These findings provide a novel pharmacological basis for the development of PNS as a potential therapeutic agent for MASLD.

## 1 Introduction

MASLD is a prevalent chronic liver condition marked by excessive lipid accumulation in hepatic tissue (Termite et al., 2025). The incidence of metabolic dysfunction-associated steatotic liver disease (MASLD) is rapidly increasing and it is now the most common chronic liver disease in world (Miao et al., 2024), affecting approximately 34% of the global population (Mignini et al., 2024). Notably, approximately 6%–30% of individuals with MASLD may progress from simple steatosis to metabolic dysfunction-associated steatohepatitis (MASH), ultimately leading to cirrhosis and hepatocellular carcinoma (Sheka et al., 2020; Mitra et al., 2020). Currently, the treatment of MASLD primarily focuses on lifestyle modifications, including weight loss, dietary changes and physical exercise. Evidence indicates that MASLD treatment may confer clinically meaningful benefits by ameliorating hepatic inflammation and promoting fibrosis regression (European Association for the Study of the Liver et al., 2024). However, few drugs are currently approved for the treatment of MASLD. Therefore, conducting research into therapeutic agents for MASLD is of paramount importance.

Oxidative stress is widely regarded as a significant contributing factor in MASLD, and is considered as a core driver in the progression from simple hepatic steatosis to MASH and fibrosis (Rolo et al., 2012). The mechanism initiates with the excessive accumulation of lipids within hepatocytes, particularly free fatty acids, which trigger the overproduction of reactive oxygen species (ROS) (Pietrocola and Bravo-San Pedro, 2021; Sheng et al., 2025), initiating a chain reaction of lipid peroxidation that generates toxic aldehydes, including malondialdehyde (MDA) and 4-hydroxynonenal (Lee et al., 2019a). These substances act as potent signaling molecules that activate key inflammatory pathways, such as nuclear factor-kappa B (Lee et al., 2019b; Machado and Diehl, 2016). Under sustained stimulation from both chronic inflammation and oxidative damage products, hepatic stellate cells are activated and differentiate into myofibroblasts, ultimately driving the onset and progression of hepatic fibrosis. Therefore, inhibiting oxidative stress may serve as a crucial therapeutic approach for MASLD.

Nrf2 is a key transcriptional activator of antioxidant response elements and a central regulator in mitigating oxidative stress. Under conditions of oxidative stress, elevated levels of ROS induce the nuclear translocation of Nrf2, which regulates downstream antioxidant genes (Smolkova et al., 2020), thereby maintaining intracellular redox homeostasis (Liu et al., 2018). For these reasons, Nrf2 pathway has been recognized as an important therapeutic target for the prevention and treatment of MASLD. As Nrf2 is a nuclear transcription factor, it does not possess intrinsic antioxidant activity; its antioxidant effects are mediated through downstream targets. In addition to well-known targets such as HO-1, metallothionein has also been identified as a potential downstream target through which Nrf2 mediates antioxidant stress responses (Gu et al., 2017). However, the mechanism by which Nrf2/metallothionein-2 (MT2) mediates antioxidant stress in MASLD remains to be fully elucidated.

Metallothionein (MT) is a class of low-molecular-weight metal-binding proteins widely expressed in multiple organisms (Thirumoorthy et al., 2007). MTs can form stable complexes with essential or toxic metal ions, such as zinc, copper, and cadmium, through thiol groups, while directly scavenging ROS and inhibiting lipid peroxidation, thereby playing a critical role in cellular antioxidant defense (Thirumoorthy et al., 2007; Ling et al., 2016; Si and Lang, 2018). Within the MT family, MT1 and MT2 are broadly expressed in various tissues, with particularly high abundance in the liver (Kimura and Kambe, 2016). Growing evidence indicates that MT2 is the predominant MT isoform in the liver and plays a crucial role in hepatic antioxidant defense. Studies have shown that the absence of MT2 significantly exacerbates HFD-induced hepatic steatosis, inflammatory infiltration, and oxidative damage markers (Wu et al., 2023). Conversely, MT2 upregulation effectively reduces oxidative damage in hepatocytes, suppresses the release of inflammatory factors, and consequently alleviates MASLD -related pathological changes (You et al., 2024). MT2 has also been considered as a key downstream antioxidant protein of Nrf2 under heavy metal exposure (Wang et al., 2025b). However, few studies have simultaneously investigated the synergistic mechanisms of Nrf2 and MT2 in MASLD, and the antioxidant roles of Nrf2/MT2 axis in MASLD development remain unclear. Therefore, the mechanisms of Nrf2 and MT2 in maintaining oxidative stress and antioxidation balance in MASLD require further elucidation.

*Panax notoginseng*, a traditional Chinese botanical drug, is derived from the dried root of *P. notoginseng* (Burkill) F. H. Chen ex C. H. Chow. Its medicinal history is long and distinguished, with Li Shizhen documenting it in his Compendium of Materia Medica during the Ming Dynasty, stating that it “can treat all blood disorders.” According to traditional Chinese medicine theory, *P. notoginseng* is believed to enter the liver and stomach meridians. It is traditionally used to disperse blood stasis, stop bleeding, reduce swelling, and alleviate pain (Xie and Wang, 2023). Modern pharmacological studies have demonstrated that *P. notoginseng* exhibits anti-hepatic fibrosis, antioxidant, anti-inflammatory, and lipid metabolism-modulating effects (Hui et al., 2016; Luo et al., 2024; Liu et al., 2020). In addition, extensive research has further expanded the therapeutic potential of *P. notoginseng* in liver diseases, such as its hepatoprotective effects against free radical induced injuries (Mancuso, 2024) and relevant to hepatic disorders through anti-inflammatory mechanisms (Kim et al., 2025). The primary bioactive components of *P. notoginseng* are a group of saponins collectively referred to as *P. notoginseng* saponins (PNS). Several studies have indicated that PNS may exert antioxidant effects through Nrf2 signaling pathway (Mi et al., 2022). However, the protective effects of studies on PNS against oxidative stress through Nrf2/MT2 axis in MASLD still need further investigation.

In this study, we hypothesized that PNS exerts protective effects against MASLD by alleviating oxidative stress and inflammation through Nrf2/MT2 axis. Accordingly, we employed an MASLD mouse model to investigate the pharmacological effects of PNS and its potential mechanisms, with a particular focus on Nrf2/MT2 pathway.

## 2 Materials and methods

### 2.1 Reagents

PNS were obtained from WuZhou Pharmaceutical CO., (#24060620b, Wuzhou, China), and rosuvastatin was purchased from AstraZeneca (#506061, AstraZeneca, London, UK). PNS were dissolved in saline at 22.5 g/L for PNS-H and 7.5 g/L for PNS-L *in vivo*. While rosuvastatin was dissolved in distilled water at 0.13 g/L. Antibodies against Nrf2 (#bs-1074R, RRID: AB_10855421), β-actin (#bsm-33139M, RRID: AB_2813873) were purchased from Bioss. Antibodies against HO-1 (#66743-1-Ig, RRID: AB_2882091) and MT2 (#ab192385, RRID: AB_3676653) were obtained from Proteintech and Abcam, respectively. Secondary antibodies, including goat anti-rabbit IgG (#SA00001-1, RRID: AB_2722565) and horse anti-mouse IgG (#7076, RRID: AB_330924), were purchased from Proteintech and Cell Signaling Technology. Notoginsenoside R1 (#80418-24-2), ginsenoside Rb1 (#41753-43-9), ginsenoside Rg1 (#22427-39-0), ginsenoside Rd (#52705-93-8), and ginsenoside Re (#52286-59-6) were obtained from Shanghai Yuanye Biological Co., Ltd (Shanghai, China), and the purity of all components was higher than 98%. MT2 protein (#URPB868Hu01) was purchased from Wuhan Yunclone Co., Ltd (Wuhan, China).

### 2.2 Animals

All animal experiments were performed on 6-8 week-old Apolipoprotein E knockout (ApoE^−/−^) male mice, purchased from MingQian Institute of Microbiology Co., Ltd (SCXK(Su)-2024-0002). Mice were housed under specific pathogen free conditions, with 12-h light/dark cycle, relative temperature 20 °C–25 °C, and relative humidity of 40%–60%, with *ad libitum* access to food and water at the Experimental Animal Center of Henan University of Chinese Medicine. All animal care and experimental procedures were approved by the Animal Experimentation Ethics Committee of Henan University of Chinese Medicine (ethics approval number: DWLLGZR202503004).

### 2.3 Mouse model of high fat diet-induced MASLD and PNS treatment

MASLD mouse model was established as previously described (Nasiri-Ansari et al., 2021). Mice in Control group were maintained on a regular chow diet, whereas mice in other groups were fed a high-fat diet (HFD: 40%–45% kcal from fat, 4.70 kcal/g, containing 0.15% cholesterol, Kuibu Shuyu Biotechnology Co.) for 6 weeks. During the HFD-fed period, mice received intraperitoneal PNS (75 mg/kg/day for PNS-L; 225 mg/kg/day for PNS-H) or oral gavage of Rosuvastatin (1.3 mg/kg/day) for 6 weeks. The dose of PNS-L used in this study was derived from clinical dose of 500 mg via intravenous administration per day, based on a human-to-mouse equivalent dose conversion ratio of 9.1, and PNS-H tripled the dose of PNS-L. The rosuvastatin dose was converted from clinical dose. Mice in control group and model group received intraperitoneal injections of an equal volume of saline. After 6 weeks of treatment, mice were anaesthetized with 0.5% pentobarbital sodium and sacrificed after fasting for 16 h. Blood samples were collected from the abdominal aorta. Blood was anticoagulated with heparin, centrifuged at 3000 rpm at 4 °C, and the plasma was collected and stored at −80 °C. The liver was excised and weighed, and divided into three parts. The first and second part were preserved in liquid nitrogen and stored at −80 °C. The third part of liver was fixed in 4% paraformaldehyde for histological analysis.

### 2.4 Biochemical analysis

The levels of triglyceride (TG), total cholesterol (TC) in liver and Low-Density Lipoprotein Cholesterol (LDL-c) in plasma were measured using quantification kits (#A110-1-1, #A111-1-1 and #A112-1-1, Nanjing Jiancheng Bioengineering Institute, Nanjing, China) according to the manufacturer’s instructions. Plasma TG and TC levels were measured using an automatic biochemical analyzer (AU400, Olympus, Tokyo, Japan). Plasma ALT and AST levels were quantified using quantification kits (#1.02.1203 and #1.02.1003, Fosun diagnostics, Shanghai, China) by an automatic biochemical analyzer.

### 2.5 Histopathological evaluation

Liver tissue was processed into paraffin sections and frozen sections, with thicknesses of 5 μm and 10 μm, respectively. For hematoxylin and eosin (HE) staining, sections were stained, images were captured by a digital slide scanner (Axioscan7, RRID: SCR_027284, Zeiss, Oberkochen, Germany), and scored according to the NAFLD activity score (NAS) protocol (Zeng et al., 2008). For Oil Red O staining, frozen liver sections were stained with 0.5% Oil Red O solution (#DL0011, Leagene, Beijing, China) for 10 min. Images were captured by digital slide scanner and the relative area of lipid droplets were quantified by ImageJ software (RRID: SCR_003070).

### 2.6 Oxidative stress determination

Liver tissue was homogenized, and protein concentrations were determined by BCA kit (#P0010, Beyotime, Shanghai, China). Hepatic levels of glutathione peroxidase (GSH-Px), superoxide dismutase (SOD) and malondialdehyde (MDA) were measured using commercial activity assay kits (#A005-1, #A001-3 and #A003-1 respectively, Nanjing Jiancheng Bioengineering Institute, Nanjing, China). Levels of GSH-Px, SOD and MDA were calculated based on the optical density of each sample at 412nm, 450 nm and 532 nm respectively using SpectraMax M2 microplate reader (RRID: SCR_020307, Molecular devices, San Francisco, California, USA) according to the manufacturer’s instructions.

### 2.7 Enzyme-linked immunosorbent assay (ELISA)

Hepatic levels of tumor necrosis factor-α (TNF-α) and interleukin-6 (IL-6) were measured using ELISA kits (#E-EL-M3063 and #E-EL-M0044, Elabscience, Wuhan, China). Liver tissue was homogenized, and protein concentrations were determined by BCA kit. Levels of TNF-α and IL-6 were calculated based on the optical density at 450 nm according to the manufacturer’s instructions.

### 2.8 RNA-sequencing analysis

RNA sequencing (RNA-seq) was performed on triplicate liver samples from mice in control, model and PNS-H groups. Total RNA was extracted using TRIzol reagent (#15596026, Invitrogen, Carlsbad, California, USA), and RNA-Seq library were constructed from 1 µg of total RNA using the VAHTS Universal V6 RNA-seq Library Prep Kit for Illumina (#NR604, Vazyme, Nanjing, China). The resulting libraries underwent quality control using a Qubit 3.0 Spectrophotometer (RRID: SCR_020311, Thermo Fisher Scientific, Waltham, Massachusetts, USA) and an Agilent 2100 Bioanalyzer (RRID: SCR_018043, Agilent Technologies, City of Santa Clara, California, USA). Following data quality filtering and sequence alignment, gene expression levels were quantified. Differentially expressed genes (DEGs) were then identified using the DESeq2. The differential analysis results were adjusted for multiple comparisons using the Benjamani–Hochberg method to control the False Discovery Rate (FDR). Genes were considered significantly differentially expressed if they met the criteria of |log2Fold Change (log2FC) | ≥ 1 and *P* < 0.05. Genes exhibiting opposite expression trends in “Model vs. Control” and “PNS-H vs. Model” comparisons were further analyzed, and the top 10 KEGG functional pathways were visualized. The top 10 enriched Gene Ontology (GO) terms in the categories of Cellular component, Molecular Function and Biological process were visualized.

### 2.9 RT-PCR

Total RNA was isolated from liver tissue using TRIzol (#15596026, Thermo Fisher Scientific, Waltham, Massachusetts, USA). Total RNA was reverse-transcribed into cDNA using the ReverTra Ace® qPCR RT Kit (#FSQ-101, TOYOBO, Osaka, Japan). Quantitative Real-time PCR was performed using the SYBR® Green Realtime PCR Master Mix (#QPK-201, TOYOBO, Osaka, Japan) on a real-Time PCR detection system (Gentier96E, Tianlong Technology, Xi’an, China). The amplification protocol was as follows: heating for 30 s at 95 °C, followed by 40 cycles of amplification (5 s at 95 °C and 30 s at 60 °C). Relative gene expression levels were calculated using the 2^−ΔΔCt method. Gene expression levels were normalized to β-actin and expressed relative to the control group. The primer sequences used in this study are listed in Table 1.

**TABLE 1:** Primers used for RT-PCR.

Gene	Sequence 5′-3′ forward	Sequences 5′-3′ reverse	Species
*ACTB*	CAT​CCG​TAA​AGA​CCT​CTA​TGC​CAA​C	ATG​GAG​CCA​CCG​ATC​CAC​A	Mouse
*Mt2*	GCC​TGC​AAA​TGC​AAA​CAA​TGC	AGC​TGC​ACT​TGT​CGG​AAG​C	Mouse
*Nfe2l2*	CTT​TAG​TCA​GCG​ACA​GAA​GGA​C	AGG​CAT​CTT​GTT​TGG​GAA​TGT​G	Mouse
*Hmox1*	AAG​CCG​AGA​ATG​CTG​AGT​TCA	GCC​GTG​TAG​ATA​TGG​TAC​AAG​GA	Mouse

### 2.10 Western blot analysis

Proteins were extracted using RIPA lysis buffer (#P0013B, Beyotime, Shanghai, China) supplemented with 1% protease inhibitor (#ST507, Beyotime, Shanghai, China). Protein concentrations were determined by BCA kit. Equal amounts of protein were separated by 8% SDS-PAGE and transferred onto polyvinylidene fluoride membranes (#IPVH00010, Merck milllipore, Darmstadt, Germany). Membranes were blocked with 5% skim milk for 1 h, and incubated overnight at 4 °C with primary antibodies against Nrf2 (1:1000), HO-1 (1:2000), MT2 (1:1000). Membranes were subsequently incubated with an HRP-conjugated secondary antibody (1:20000) at room temperature for 1 h. Protein bands were visualized using an enhanced chemiluminescence detection system (Touch Imager, e-BLOT, Shanghai, China), and band intensities were quantified using ImageJ software.

### 2.11 Immunohistochemical staining

Paraffin-embedded tissue sections were deparaffinized in xylene. Endogenous peroxidase was blocked. After blocking with 10% goat serum for 30 min, sections were incubated at 4 °C overnight with a primary antibody against MT2 (1:100). Sections were then incubated with a reaction enhancer (#PV-9001, ZSbio, Wuhan, China) for 20 min at 37 °C. Subsequently, sections were washed with phosphate-buffered saline and incubated with HRP-conjugated secondary antibody for 1 h at 37 °C. Immunoreactivity was visualized using diaminobenzidine, followed by counterstaining with haematoxylin. Representative images were captured using a digital slide scanner.

### 2.12 Molecular docking

The two-dimensional structures (SDF format) of notoginsenoside R1, ginsenoside Rb1, ginsenoside Rg1, ginsenoside Rd, and ginsenoside Re were downloaded from the PubChem database (https://pubchem.ncbi.nlm.nih.gov/) as ligand molecules. The three-dimensional crystal structure of MT2 (PDB ID 4MT2) was retrieved from the Protein Data Bank (PDB) as the receptor structure. The receptor structure was prepared by removing water molecules and co-crystallized ligands, while ligand structures optimized using the RDKit toolkit (RRID: SCR_014274). Apply modifications using AutoDockTools software (RRID: SCR_026401). Molecular docking between the receptor and ligands was performed using AutoDock Vina (RRID: SCR_011958). Binding sites were automatically identified by the program, and 10 docking runs were performed for each ligand. The conformation with the lowest binding free energy was selected. Docking interactions were visualized using Discovery Studio Visualizer 2019 software (RRID: SCR_008398).

### 2.13 Surface plasmon resonance

SPR measurements were performed on a Biacore instrument (T200, RRID: SCR_019718, Cytiva, Uppsala, Sweden). The sensor chip surface (flow cell 4) was activated using 1-ethyl-3- (3-dimethylaminopropyl) carbodiimide (EDC, GE Healthcare) and N-hydroxysuccinimide (NHS, GE Healthcare) at a flow rate of 10 μL/min for 420 s. MT2 protein (ligand) was diluted with pH 4.5 sodium acetate to a final concentration of 10 μg/mL and the protein was fixed in the 4-channel of the chip at a flow rate of 10 μL/min. The coupling reaction was allowed to proceed for 100 s, and successful immobilization was confirmed by the sensorgram response. A series of concentrations were injected onto the MT2-immobilized surface to determine binding affinities: 62.5, 31.25, 15.63, 7.81, 3.91, and 0 μM for notoginsenoside R1; 1000, 500, 250, 125, 62.5, 31.25, 15.63, 7.81, 3.91, 1.95, and 0 μM for ginsenoside Rb1; 500, 250, 125, 62.5, 31.25, 15.63, and 0 μM for ginsenoside Rg1; and 12.5, 6.25, 3.13, 1.56, 0.78, 0.39, and 0 μM for ginsenoside Rd and Re. Sample data was collected using Biacore T200 control software and reference flow cell responses was subtracted for background correction. The data were collected using Biacore T200 software (RRID: SCR_019718) to calculate the association rate constant (ka), dissociation rate constant (kd), and equilibrium dissociation constant (KD).

### 2.14 Statistical analysis

The data were analyzed using the SPSS23.0 statistical software (RRID: SCR_002865) and expressed as the mean ± SEM. Comparisons between groups were compared with a single-factor analysis of variance (one-way ANOVA) or the nonparametric Kruskal-Wallis test for non-normally distributed data. *P* < 0.05 was considered statistically significant differences.

## 3 Results

### 3.1 Chemical quality control of PNS

PNS used in this study is composed of ginsenoside Rg1 (46.1%), ginsenoside Rb1 (25.4%), notoginsenoside R1 (13.8%), ginsenoside Re (6.4%), and ginsenoside Rd (1.71%). The analysis report was provided by Wuzhou Pharmaceutical Group (Supplementary Figures S1). The total content of the five main components exceeds 90%, with trace saponins accounting for less than 10%, which was consistent with previous report (Yang et al., 2016).

### 3.2 PNS alleviated hepatic damage in mice fed a HFD along with improvement of hepatic steatosis

**FIGURE 1:**
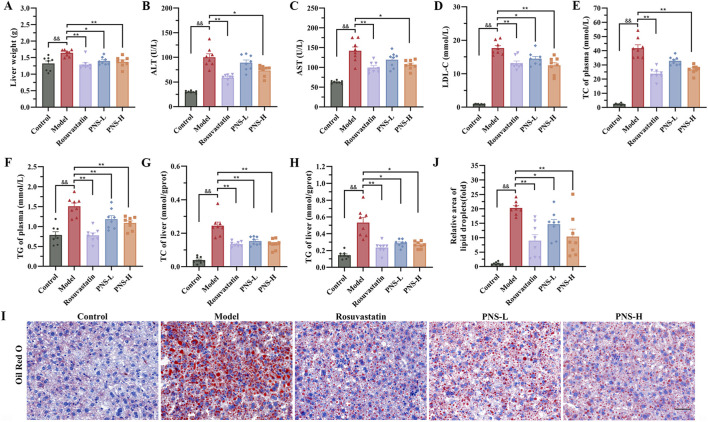
PNS alleviates lipid accumulation and improves liver function in MASLD mice. **(A)** Liver weight of mice in each group. **(B,C)** Levels of ALT and AST in plasma. **(D–F)** Levels of LDL-c, TC and TG in plasma. **(G,H)** Levels of TC and TG in liver tissue. **(I)** Representative Oil Red O staining images of liver tissue (400×, scale bar: 25 μm). **(J)** Quantitative analysis of the relative area of lipid droplets in Oil Red O-stained sections. n = 8. Data are presented as mean ± SEM. *&&P* < 0.01 vs. Control group; *P < 0.05, **P < 0.01 vs. Model group.

In our study, liver weight was significantly increased in model group compared with control group (Figure 1A), while PNS treatment significantly attenuated this increase. Concurrently, levels of ALT, AST, LDL-c, TC, and TG in plasma were markedly elevated in model group (Figures 1B–F), indicating impaired liver function and disrupted lipid metabolism. Compared with model group, PNS-H significantly reduced plasma ALT, AST and plasma TC levels, while both PNS-L and PNS-H decreased plasma LDL-c and TG levels. Additionally, compared with control group, hepatic TC and TG levels were significantly increased in model group (Figures 1G,H). Both PNS-L and PNS-H significantly suppressed hepatic TC and TG levels, compared with model group.

Oil Red O staining further demonstrated marked lipid droplets accumulation in liver tissue of model group, compared with the control group (Figure 1I). Both PNS-L and PNS-H markedly reduced hepatic lipid droplet area in a dose-dependent manner, with a reduction rate of approximately 30% and 55%, respectively, compared with model group. These findings suggest that PNS exerts protective effects against MASLD by regulating lipid levels, reducing hepatic lipid accumulation, and improving liver function.

### 3.3 PNS demonstrated therapeutic efficacy against hepatic oxidative stress and inflammation in HFD-induced MASLD mice

**FIGURE 2:**
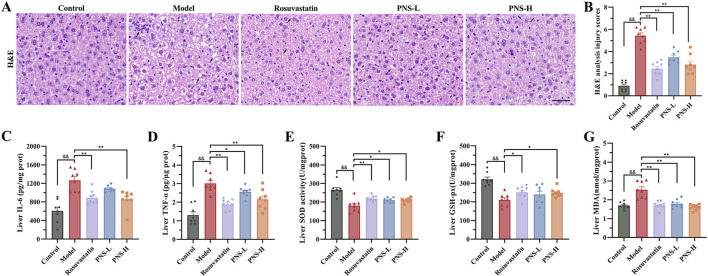
PNS ameliorates liver histological injury and oxidative stress in MASLD mice. **(A)** Representative H&E staining images of liver tissue (400×, scale bar: 50 μm). **(B)** Quantitative analysis of liver injury scores based on NAS. **(C,D)** The levels of inflammatory factors IL-6 and TNF-α. **(E–G)** Hepatic activities of SOD, GSH-Px, and MDA. n = 8. Data are presented as mean ± SEM. *&&P* < 0.01 vs. Control group; *P < 0.05, **P < 0.01 vs. Model group.

Histopathological analysis revealed that, compared with control group, severe hepatic steatosis, ballooning degeneration, and inflammatory infiltration were observed in model group, whereas these HFD-induced pathological changes were markedly attenuated by PNS treatment (Figure 2A). NAS evaluation also revealed that the NAS score was significantly increased in model group (5.43 ± 0.25) compared with control group, whereas both PNS-L and PNS-H group significantly reduced the NAS score (3.48 ± 0.19 and 2.83 ± 0.30, respectively) (Figure 2B), indicating hepatoprotective effects of PNS in HFD-induced MASLD mice.

Compared with control group, hepatic levels of the proinflammatory factors IL-6 and TNF-α were significantly elevated in model group, PNS-H significantly reduced hepatic levels of IL-6, and both PNS-L and PNS-H markedly reduced hepatic levels of TNF-α (Figures 2C,D). The activities of SOD and GSH-Px were significantly decreased in model group, whereas hepatic MDA levels were significantly increased. Compared with model group, PNS-H significantly increased activities of GSH-Px, and both PNS-L and PNS-H markedly increased activities of SOD, and significantly decreased hepatic MDA levels (Figures 2E–G). These findings collectively indicate that PNS exerts significant anti-inflammatory and antioxidant effects in MASLD mice.

### 3.4 Transcriptomics reveals PNS attenuates oxidative damage, potentially via modulation of Nrf2 and MT2 in MASLD

**FIGURE 3:**
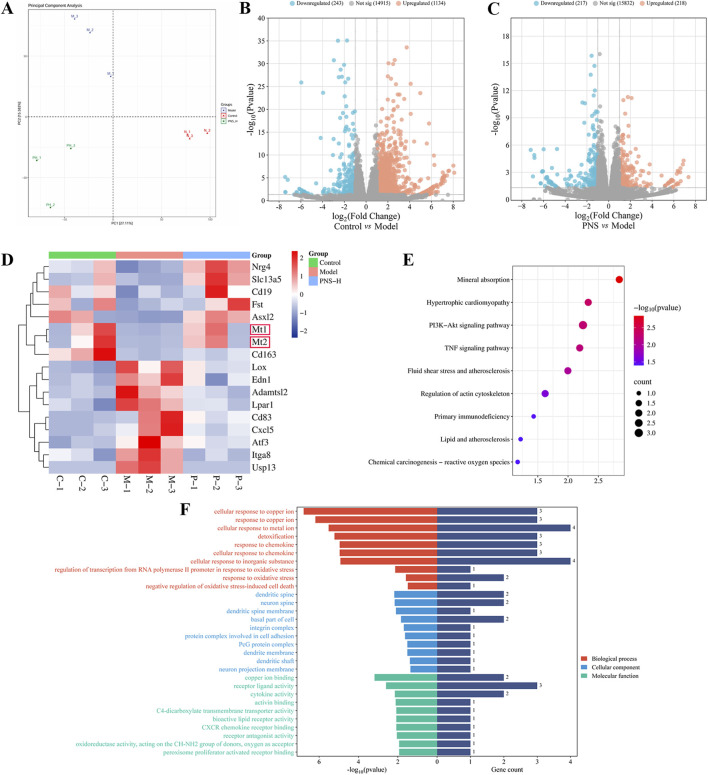
Transcriptomic analysis reveals that PNS mediates oxidative stress and lipid metabolism via Nrf2/MT2 pathway. **(A)** Principal component analysis of liver transcriptomes from Control, Model, and PNS-H groups. **(B,C)** Volcano plots of differentially expressed genes (DEGs) in Control vs. Model and PNS-H vs. Model. **(D)** Heatmap of co-existing DEGs among three groups. **(E,F)** KEGG and GO enrichment analysis for co-existing DEGs among three groups. n = 3.

To investigate the mechanism by which PNS mitigates MASLD, liver tissue of control group, model group and PNS-H group were subjected to transcriptomics analysis. 1,134 genes were upregulated and 243 were downregulated in model group relative to control group (Figures 3B,C). Between model group and PNS group, 218 genes were upregulated and 217 were downregulated. A clustering heatmap of co-existing differentially expressed genes across the three groups was generated, revealing that both MT1 and MT2 were significantly downregulated in model group, while their expression levels were restored toward normalization following PNS treatment (Figure 3D). KEGG pathway enrichment analysis indicated that these differentially expressed genes were significantly enriched in oxidative stress and inflammation-related pathways including the “TNF signaling pathway” and “Chemical carcinogenesis-reactive oxygen species” (Figure 3E).

GO enrichment analysis was subsequently performed (Figure 3F), and functional annotation confirmed that these genes are predominantly involved in “cellular response to copper ions” and “oxidative stress response”. Collectively, these findings indicated that MASLD significantly activated pathways associated with oxidative stress and inflammation, and PNS treatment alleviated oxidative damage and ameliorates related pathological processes, potentially through modulation of MT2.

### 3.5 PNS intervention activates antioxidant pathways by upregulating the expression of MT2, Nrf2, and HO-1

**FIGURE 4:**
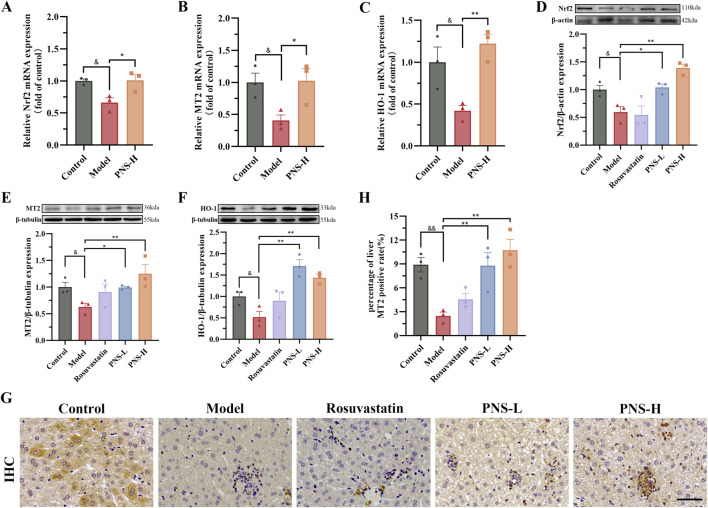
PNS upregulates Nrf2/MT2 expression in MASLD mice. **(A–C)** Hepatic mRNA expression levels of Nrf2 **(A)**, MT2 **(B)**, and HO-1 **(C)**. **(D–F)** Hepatic Nrf2, MT2, and HO-1 protein expression. **(G)** Representative IHC staining images of MT2 in liver sections (400X, scale bar: 50 μm). **(H)** Quantitative analysis 8 visual fields. n = 3. Data are presented as mean ± SEM. *&P* < 0.05, *&&P* < 0.01 vs. Control group; *P < 0.05 vs. Model group.

To further verify whether PNS regulates MT2 through Nrf2 to exert an anti-oxidative stress effect, the expression of oxidative stress-related mRNA was analyzed, revealing that, in model group, mRNA expression of Nrf2, MT2, and HO-1 were significantly downregulated relative to control group (Figures 4A–C). After PNS treatment, mRNA levels of all three genes were significantly upregulated. WB results further confirmed that protein expression of Nrf2, MT2 and HO-1 were significantly downregulated in model group, compared with control group. Following PNS treatment, both PNS-L and PNS-H significantly increased the expression of Nrf2, MT2, and HO-1 (Figures 4D–F). Furthermore, immunohistochemical analysis (Figure 4G) demonstrated that MT2 was highly co-localized with inflammatory infiltration in liver tissue, and expression of MT2 was markedly upregulated in PNS-H group, compared with model group (Figure 4H). In summary, PNS confers hepatoprotective effects potentially by activating Nrf2 signaling pathway, thereby upregulating downstream antioxidant target proteins, including MT2 and HO-1.

### 3.6 The five major saponins of PNS exhibit molecular docking affinity with MT2

**FIGURE 5:**
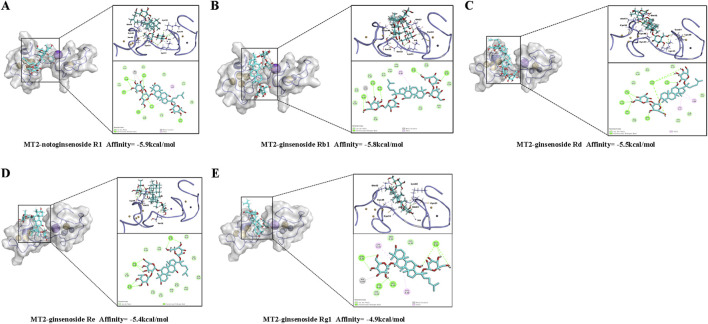
Major saponins of PNS exhibit binding conformations with MT2. **(A)** Notoginsenoside R1 had a binding affinity of −5.9 kcal/mol to MT2 receptor; **(B)** Ginsenoside Rb1 had a binding affinity of −5.8 kcal/mol to MT2 receptor; **(C)** Ginsenoside Rd had a binding affinity of −5.5 kcal/mol to MT2 receptor; **(D)** Ginsenoside Re had a binding affinity of −5.4 kcal/mol to MT2 receptor; **(E)** Ginsenoside Rg1 had a binding affinity of −4.9 kcal/mol to MT2 receptor. Cd ions manifest as yellow, Zn manifests as dark blue, and Na ions manifest as purple.

Molecular docking analysis revealed that the five major saponins of PNS exhibited favorable binding conformations with MT2 protein. Free energy calculations showed that notoginsenoside R1 formed hydrogen bonds with Lys22, Cys24, Lys31, Cys33, Ser45, and Glu52 on the MT2 protein, while establishing hydrophobic interactions with Cys33 and Cys48 and a metal-ion interaction with Na^+^, resulting in a binding energy of −5.9 kcal/mol (Figure 5A). Ginsenoside Rb1 formed hydrogen bonds with Gln23, Cys24, Ser32, Lys31, and Ser35 on MT2, along with hydrophobic interaction with Cys48 and a metal-ion interaction with Na^+^, with a binding energy of −5.8 kcal/mol (Figure 5B). Ginsenoside Rd formed hydrogen bonds with Lys20, Cys21, Lys31, Cys50, Glu52, and Ala52 of MT2, and hydrophobic interactions with Cys33 and Cys48, resulting in a binding energy of −5.5 kcal/mol (Figure 5C). Ginsenoside Re formed hydrogen bonds with Ser32, Cys48, and Glu52 of MT2, with a binding energy of −5.4 kcal/mol (Figure 5D). Ginsenoside Rg1 formed hydrogen bonds with Cys21, Lys22, Gln23, and Glu52 on the protein, while also forming hydrophobic interactions with Cys33 and Cys48 of MT2, and a metal-ion interaction with Na^+^, yielding a binding energy of −4.9 kcal/mol (Figure 5E).

### 3.7 Ginsenoside Rd displays the most stable binding conformation to MT2 among major saponins of PNS

**FIGURE 6:**
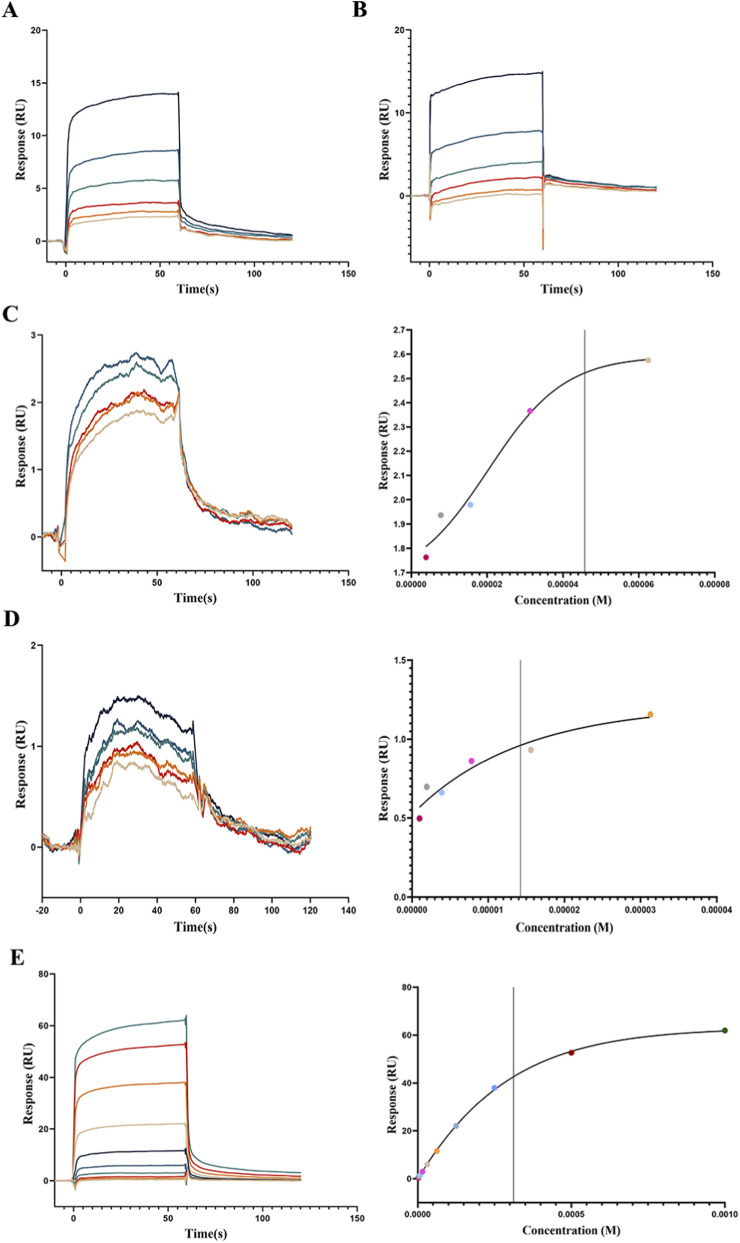
SPR validates strong binding of ginsenoside Rd, ginsenoside Rg1, notoginsenoside R1 and ginsenoside Re to MT2. **(A)** The binding and dissociation curves between ginsenoside Rd and MT2. **(B)** The binding and dissociation curves between ginsenoside Rg1 and MT2. **(C)** The binding and dissociation curves between notoginsenoside R1 and MT2. **(D)** The binding and dissociation curves between ginsenoside Re and MT2. **(E)** The binding and dissociation curves between ginsenoside Rb1 and MT2.

Based on the findings from molecular docking, SPR experiment was proceeded to validate the molecular interaction between five major saponins of PNS and MT2 protein. The dissociation constant (KD) describes the strength of the binding between the ligand and the analyzing molecule. The SPR data indicated that KD between ginsenoside Rd (Figure 6A), ginsenoside Rg1 (Figure 6B), notoginsenoside R1 (Figure 6C), ginsenoside Re (Figure 6D), ginsenoside Rb1 (Figure 6B) and MT2 protein were 3.040 × 10^−6^ M, 3.047 × 10^−5^ M, 4.574 × 10^−5^ M, 1.432 × 10^−5^ M and 3.124 × 10^−4^ M, respectively. The association rate constant (Ka) between ginsenoside Rd, ginsenoside Rg1 and MT2 protein were 1.251 × 10^4^ 1/M and 0.585 × 10^3^ 1/M, respectively. And the dissociation rate constant (Kd) between ginsenoside Rd, ginsenoside Rg1 and MT2 protein were 1.672 × 10^−2^ 1/s and 3.804 × 10^−2^ 1/s, respectively. These results demonstrate that ginsenoside Rd may have the most stable binding conformation with MT2.

## 4 Discussion

The onset and progression of MASLD are driven by multiple molecular mechanisms, with the core pathological basis being lipid metabolism imbalance (Nie et al., 2023). To evaluate the therapeutic efficacy of PNS against MASLD, this study employed ApoE^−/−^ mice combined with HFD to establish a MASLD model. The MASLD model exhibited significant increases of liver weight, TC, TG, LDL-c, as well as ALT and AST levels. These findings are consistent with previous studies and collectively reflect hallmark features of lipid metabolism disorders and hepatic injury (Im et al., 2021; Wu et al., 2022). PNS intervention exerted significant ameliorative effects against MASLD. PNS effectively normalized the elevated lipid metabolism indicators and hepatic injury markers. Moreover, PNS markedly attenuated hepatic histological lesions. Collectively, these findings suggest that PNS may exert multi-targeted protective effects in MASLD progression by regulating lipid metabolism and suppressing inflammatory responses.

ApoE^−/−^ mice were selected in this study, ApoE^−/−^ mice exhibit severe disruption of basal lipid metabolism due to the absence of apolipoprotein E, characterized by markedly elevated plasma cholesterol levels and impaired lipoprotein clearance (Knowles and Maeda, 2000; Huebbe et al., 2024). In this genetic background, HFD intervention rapidly and reliably induces characteristic MASLD pathological changes, including significant hepatic steatosis, ballooning degeneration, inflammatory infiltration, and progression to steatohepatitis and early-stage fibrosis (Camargo et al., 2022; Huang et al., 2022). In contrast, C57BL/6 mice fed a HFD alone may develop obesity, yet their hepatic lesions confined to simple steatosis. Such lesions rarely progress spontaneously to stages marked by severe inflammation and fibrosis, which represent hallmark features of disease progression in clinically diagnosed MASLD patients (Flessa et al., 2022; Oligschlaeger and Shiri-Sverdlov, 2020).

Previous studies have shown that major saponins of PNS potentially exert hepatoprotective effects through Nrf2 pathway. Ginsenoside Rd was reported to inhibit lipid peroxidation and exert antioxidant effects via Nrf2 pathway in metabolism associated fatty liver disease (Liu, W., et al., 2025). Study further demonstrated that ginsenoside Rd significantly reduced ROS levels, lipid peroxide levels, and mitochondrial stress in hepatocytes (Cui, T., et al., 2023). Notoginsenoside R1 has also been reported to activate the Nrf2/ARE signaling pathway, thereby increase the expression of antioxidant enzymes (Wang, Y., et al., 2023b). Other ginsenosides including ginsenoside Rb1, ginsenoside Rg1, and ginsenoside Re have also been demonstrated to exert hepatoprotective effects through multiple signaling pathways (Jin et al., 2023). Our study further demonstrated that PNS alleviates oxidative stress in MASLD potentially through Nrf2/MT2. In addition to regulating MT2 expression, we found that ginsenoside Rd exhibits favorable binding to MT2 with a KD of 3.04 μM. This direct interaction may complement the transcriptional upregulation of MT2, providing a more comprehensive mechanistic basis for the protective effects of PNS.

The development of MASLD is complex and driven by multiple factors. According to the “multiple-hit” hypothesis, pathogenic factors, such as insulin resistance, dietary components, epigenetics, genetics, and the gut microbiota, may collectively contribute to the advancement of simple steatosis toward MASH (Wang et al., 2024). Among them, oxidative stress serves as the key mediator by which all the previously mentioned pathogenic factors converge, ultimately resulting in hepatocellular death and tissue damage (Termite et al., 2025). Under HFD conditions, ROS accumulate extensively in the liver, inducing lipid peroxidation, mitochondrial dysfunction, and inflammatory signaling cascades, thereby promoting disease progression. Concurrently, chronic inflammation induced by oxidative stress constitutes a critical driver of hepatocyte injury, hepatic fibrosis, and the progressive worsening of the disease. In this study, pathways related to oxidative stress and metal ion metabolism were significantly enriched in MASLD model. Consequently, attention was directed toward Nrf2 signaling pathway and its potential downstream molecule MT2. As a transcriptional activator of the antioxidant response, Nrf2 knockdown experiments have demonstrated that Nrf2 plays a key role in regulating MT2 expression. Study have shown that Nrf2 was recruited to the antioxidant response element (ARE) of the promoter region of the MT2 gene in vascular endothelial cells (Shinkai et al., 2016). Furthermore, using Nrf2 knockout mice, it has also been shown that Nrf2 is indispensable for the transcriptional upregulation of MT2 in diabetic cardiomyopathy (Gu et al., 2017). In this study, PNS treatment reduced plasma TNF-α and IL-6 levels, decreased MDA content and enhanced hepatic GSH-Px and SOD activities. Transcriptomics and KEGG pathway analysis further indicated that PNS may ameliorate oxidative stress and chronic inflammation in MASLD by regulating MT2. There are various studies have reported the regulatory effect of PNS on the Nrf2 signaling pathway. It has been shown that PNS activate Nrf2/ARE pathway and ameliorates hepatic damage induced by triptolide (Feng et al., 2019). Moreover, activation of the Nrf2 signaling pathway has been demonstrated to mediate the protective effects of PNS against oxidative stress in blood-brain barrier disruption (Hu et al., 2018). Our findings demonstrate that PNS markedly upregulate Nrf2 expression, thereby upregulates MT2 expression in MASLD. The synergistic upregulation of Nrf2 and MT2 indicates that, MT2 likely serves as a novel downstream effector of Nrf2 pathway and potentially regulated by PNS in MASLD.

Molecular docking analysis revealed that the five major saponins of PNS directly bind to the MT2 protein. These interactions with MT2 involve multiple modes, including hydrogen bonds, hydrophobic interactions, and metal coordination. Subsequent SPR validation demonstrated that ginsenoside Rd displayed favorable binding with MT2. In contrast, notoginsenoside R1, ginsenoside Re and ginsenoside Rb1 displayed much weaker binding affinity. Structurally, five major saponins of PNS belong to two distinct classes: ginsenoside Rd, ginsenoside Rb1 and notoginsenoside R1 are protopanaxadiol (PPD)-type saponins, whereas ginsenoside Rg1 and ginsenoside Re belong to the protopanaxatriol (PPT)-type. Among the PPD-type saponins, ginsenoside Rd and notoginsenoside R1 each carry three glycosides, while ginsenoside Rb1 has four glycosides. The presence of an additional terminal glucose at the C-20 position of ginsenoside Rb1 likely introduces significant steric hindrance, which may prevent optimal accommodation into the MT2 binding pocket. Furthermore, although both ginsenoside Rd and notoginsenoside R1 are PPD-type saponins with three glycosides, they differ in the glycosidic substitution at the C-3 position: ginsenoside Rd has a β-D-Glc-(1→2)-β-D-Glc substituent, while notoginsenoside R1 has an α-D-Xyl-(1→2)-β-D-Glc substituent. The replacement of the inner glucose with xylose in notoginsenoside R1 may reducing complementary interactions with MT2. Collectively, these observations suggest that the binding affinity of PNS saponins to MT2 is determined by a combination of factors, including the saponin class, the number of glycosides, the composition of the sugar chain at key positions, and the resulting steric and conformational properties. Previous investigations have concentrated on the transcriptional upregulation of MT2. For example, pravastatin improved the epithelial barrier in radiation-induced enteropathy through upregulation of MT2 (Kwak et al., 2021), and quercetin upregulates MT2 expression and exerts antioxidant effects in environmental oxidant-induced liver damage (Weng et al., 2011). However, several small molecules have been reported to directly bind to MT2. Vitamin C has been shown to directly binds to MT2, thereby enhancing its antioxidant and neuroprotective effects (Hassan et al., 2024), and a recent study showed that Artesunate provided neuroprotection against Parkinson’s disease via directly binding and upregulation of MT2A, decreased intracellular Cu^2+^ level and improved dopamine neuronal cuproptosis (Cheng et al., 2025). Similarly, our findings suggest that ginsenoside Rd may interact directly with MT2 to maintain metal homeostasis, thereby exerting antioxidant effect.

Chronic inflammation is widely recognized as a pivotal driver of MASLD progression. In this study, inflammatory mediators were significantly elevated in MASLD model (Mo et al., 2025; Xu et al., 2021), following PNS intervention, hepatic TNF-α and IL-6 levels were markedly reduced. Immunohistochemical analysis further revealed significantly upregulated MT2 expression in PNS-treated groups within areas of inflammatory infiltration. These finding suggest that PNS may exerts anti-inflammatory effects by modulating MT2-mediated antioxidant pathways, thereby interrupting a critical step in the progression from MASLD to NASH. Nevertheless, several issues remain to be clarified. Firstly, to determine whether PNS regulates MT2 via an Nrf2-dependent pathway and elucidate its role in MASLD, future studies could employ Nrf2 knockout models for validation. Secondly, recent studies indicate that MT2 can inhibit iron-dependent lipid peroxidation and copper-mediated mitochondrial toxicity by chelating free iron and copper ions, thereby exerting a core protective function in two novel cell death pathways: ferroptosis and cuproptosis (Yu et al., 2024; Wang et al., 2025a). Therefore, building upon the clarification of PNS-mediated regulation of MT2, future studies may investigate hepatocyte death patterns in the context of MASLD. The focus should be on elucidating whether PNS modulates iron and copper ion homeostasis within hepatocytes via MT2 upregulation, thereby inhibiting ferroptosis and cuproptosis. Thirdly, in this study, our results do not clarify whether PNS directly activates Nrf2 or acts through its upstream regulator such as Keap1. Future studies employing Keap1-knockdown or Nrf2-knockout models will help further elucidate whether PNS directly activates Nrf2 or acts through Keap1.

## 5 Conclusion

Our study demonstrates that PNS effectively reduces oxidative stress and inflammation in MASLD, potentially through Nrf2/MT2 pathway, and ginsenoside Rd exhibits favorable binding to MT2. Collectively, these findings indicate that PNS may serve as a potential therapeutic candidate for ameliorating MASLD.

## Data Availability

The data presented in the study are deposited in the NCBI repository, accession number PRJNA1462623.
