# Supplementary material for: Panax notoginseng saponins alleviates metabolic dysfunction-associated steatotic liver disease in mice by influencing Nrf2/MT2
Source: Front Pharmacol. 2026 Jun 29;17:1874424. doi: 10.3389/fphar.2026.1874424 (PMC13382251; doi:10.3389/fphar.2026.1874424)
Supplement: Supplementary file 1 [file Supplementaryfile1.docx]

Supplementary Material

## Supplementary Figure 1


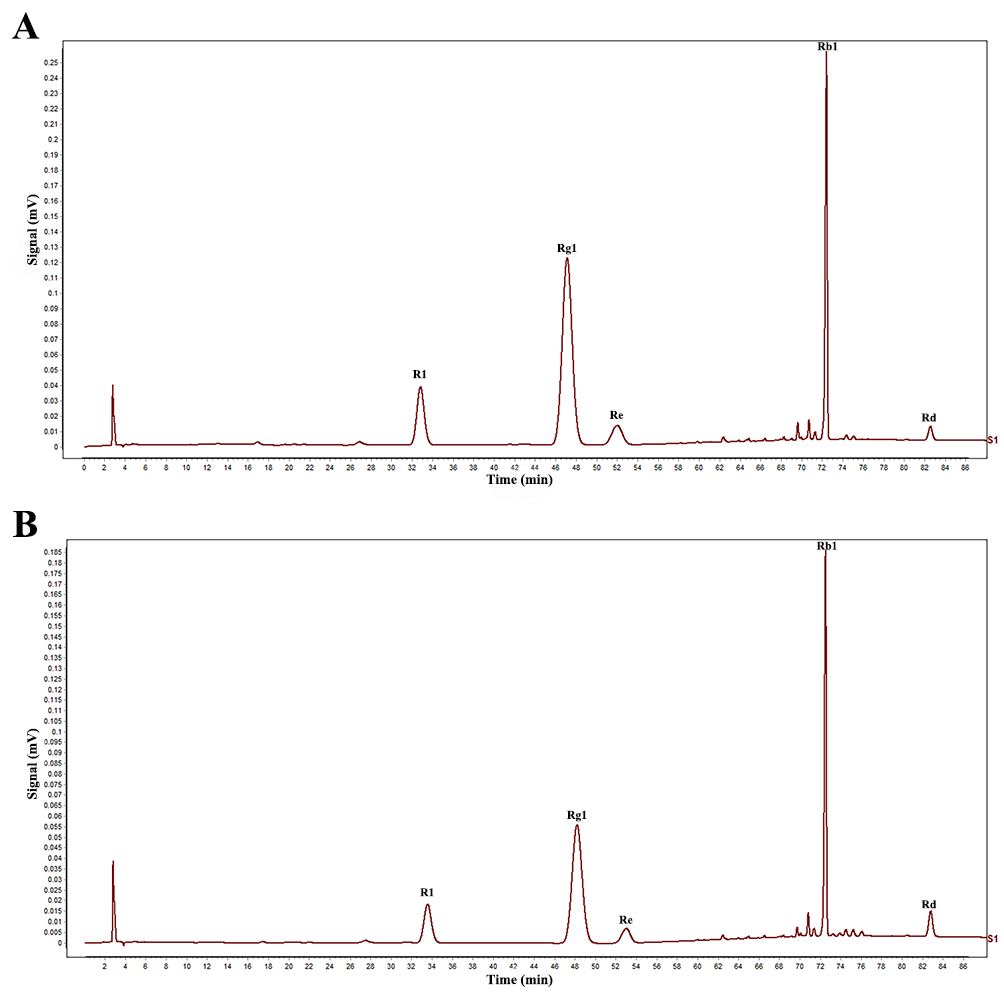


**Supplementary Figure 1.** **Chemical quality control of PNS.** (A) Chemical analysis data of PNS provided by Wuzhou Pharmaceutical Group. (B) Chemical analysis data of Reference standards.
